# Age-Related Differences in the Mouse Corneal Epithelial Transcriptome and Their Impact on Corneal Wound Healing

**DOI:** 10.1167/iovs.65.5.21

**Published:** 2024-05-13

**Authors:** Anmar Abu-Romman, Kaitlin K. Scholand, Gowthaman Govindarajan, Zhiyuan Yu, Sonali Pal-Ghosh, Mary A. Stepp, Cintia S. de Paiva

**Affiliations:** 1 Ocular Surface Center, Cullen Eye Institute, Department of Ophthalmology, Baylor College of Medicine, Houston, Texas, United States; 2 Department of Biosciences, Rice University, Houston, Texas, United States; 3 Department of Anatomy and Cell Biology, The George Washington University School of Medicine and Health Sciences, Washington, DC, United States; 4 Department of Ophthalmology, The George Washington University School of Medicine and Health Sciences, Washington, DC, United States

**Keywords:** cornea, aging, wound debridement, epithelial wound healing, re-epithelialization, cornea sensitivity, molecular pathways, epithelial erosions

## Abstract

**Purpose:**

Aging is a risk factor for dry eye. We sought to identify changes in the aged mouse corneal epithelial transcriptome and determine how age affects corneal sensitivity, re-epithelialization, and barrier reformation after corneal debridement.

**Methods:**

Corneal epithelium of female C57BL/6J (B6) mice of different ages (2, 12, 18, and 24 months) was collected, RNA extracted, and bulk RNA sequencing performed. Cornea sensitivity was measured with an esthesiometer in 2- to 3-month-old, 12- to 13-month-old, 18- to 19-month-old, and 22- to 25-month-old female and male mice. The 2-month-old and 18-month-old female and male mice underwent unilateral corneal debridement using a blunt blade. Wound size and fluorescein staining were visualized and photographed at different time points, and a re-epithelialization rate curve was calculated.

**Results:**

There were 157 differentially expressed genes in aged mice compared with young mice. Several pathways downregulated with age control cell migration, proteoglycan synthesis, and collagen trimerization, assembly, biosynthesis, and degradation. Male mice had decreased corneal sensitivity compared with female mice at 12 and 24 months of age. Aged mice, irrespective of sex, had delayed corneal re-epithelialization in the first 48 hours and worse corneal fluorescein staining intensity at day 14 than young mice.

**Conclusions:**

Aged corneal epithelium has an altered transcriptome. Aged mice regardless of sex heal more slowly and displayed more signs of corneal epithelial defects after wounding than young mice. These results indicate that aging significantly alters the corneal epithelium and its ability to coordinate healing.

The cornea is the avascular and transparent anterior part of the eye with significant refractive and barrier protective functions. It is covered with a tear film that, together with the underlying cornea, protects the eye from environmental and microbial damage. Most of the corneal epithelial cells turnover between 7 and 14 days after migrating onto the corneal surface from the limbus, but a small number of cells have been shown to remain on the cornea for up to 4 months.^1^^,^^2^

Rapid and efficient re-epithelialization of the injured cornea is essential. Impaired corneal epithelial migration increases the risk of infection in the eye, leads to edema and loss of corneal transparency, and decreases quality of life.^3^^,^^4^ Compared with thermal and chemical injuries, the most common form of injury to the epithelium is mechanical.^5^ Rapid healing after corneal debridement is achieved by altering adhesion between the epithelial cells and the basement membrane, disassembling hemidesmosomes, and cells transitioning from a quiescent to an activated phenotype.^6^^–^^11^ These changes are mediated by exposure of the corneal epithelial cells to growth factors and cytokines in the tears and to factors secreted by corneal epithelial, resident, and recruited immune cells.^9^^–^^13^ Within 6 hours, leader corneal epithelial cells emerge at the wound edge to direct migration of the epithelial sheet over the exposed substrate.^14^ Sheer injuries leave the basement membrane intact; cells migrate over a laminin- and proteoglycan-rich basement membrane.^15^^,^^16^ Deeper injuries expose migrating cells to the collagens and proteoglycans that comprise the stroma. As re-epithelialization proceeds, the numbers of cell layers in the migrating corneal epithelial sheet decrease.^17^^,^^18^ Once complete, tight junctions reassemble and begin to restore the barrier function of the corneal epithelium.^19^ Epithelial cell proliferation then restores the number of cells and cell layers in the corneal epithelium.^20^ Finally, the corneal epithelial basal cells direct the reassembly of the hemidesmosomes and resynthesis of the epithelial basement membrane.^21^ Deeper stromal injuries activate stromal fibroblasts to produce and assemble stromal collagens and proteoglycans, as well as some components of the epithelial basement membrane.^22^ These events take place beneath an intact corneal epithelium after re-epithelialization is complete. If basement membrane reassembly is delayed, the epithelial sheet will be prone to detachment, leading to erosion formation.^23^^,^^24^

The cornea is innervated by the ophthalmic branch of the trigeminal nerve. In the corneal epithelium, intraepithelial corneal nerves terminate into thousands of nerve endings, leading to high sensitivity to foreign chemicals, objects, and changes in temperature.^25^^,^^26^ Corneal nerves produce neuropeptides that promote corneal epithelial homeostasis and stimulate wound closure.^27^^–^^29^ Events that positively or negatively impact corneal epithelial cells also influence the intraepithelial nerves.

Studies have shown that the inflammation that accompanies aging is harmful to many tissues.^30^^–^^33^ Inflammatory and other age-related changes have also been reported on the ocular surface and cornea.^34^^,^^35^ We and others have used aged mice as a model of dry eye disease. As seen in dry eye disease, aged mice have (1) increased corneal permeability,^36^^–^^38^ (2) conjunctival goblet cell loss,^37^^,^^39^^,^^40^ (3) increased corneal irregularity,^41^ (4) increased immune infiltration in the conjunctiva,^42^ (5) meibomian gland disease,^43^^–^^48^ (6) an altered tear immunoglobulin profile,^49^ (7) altered tear cytokines,^40^^,^^50^^,^^51^ and (8) increased immune infiltration in the lacrimal gland.^36^^,^^48^^,^^50^^–^^54^ Corneal aging produces both structural and functional changes that can affect the ability of the organ to refract light, repair itself, and maintain its barrier function.^55^^,^^56^ Previously, we have reported on a decrease in intraepithelial corneal nerve density and cornea sensitivity accompanying aging in mice.^41^ We identified an age-related decrease in specific genes involved in encoding proteins that mediate axon growth and targeting in the corneal epithelium.^41^ However, the molecular pathways involved in the maintenance of corneal epithelium and the effects of aging on cornea wound healing have not been investigated thoroughly.

The purpose of this study was two-fold. First, we investigated molecular pathways and transcriptome changes within the corneal epithelium as mice age. Among the pathways we found altered in aged mice were those that mediate cell:matrix adhesion and re-epithelialization. After assessment of age- and sex-dependent differences in corneal sensitivity, our second objective was to evaluate corneal wound healing in young and aged male and female mice.

## Methods

### Animals

All animal studies performed were conducted following the approval of The Institutional Animal Care and Use Committees at Baylor College of Medicine's Center for Comparative Medicine and Institutional Review Board. Furthermore, all studies conformed to the standards in the ARVO Statement for the Use of Animals in Ophthalmic and Vision Research.

The experiments were performed at the Ocular Surface Center in Baylor College of Medicine (Houston, TX, USA). C57BL/6J (B6) mice of both sexes were used. These mice were either purchased from Jackson Laboratories (stock 000664, Jackson Laboratories, Bar Harbor, ME, USA), aged in house, or were received from the National Institute of Aging. All mice were housed in a specific pathogen-free vivarium managed by Baylor College of Medicine Center for Comparative Medicine. Several age groups were used: 2 to 3 months (35 females and 29 males; referred to as the young group in text and figure legends); 12 to 13 months (30 females and 10 males); 18 to 19 months (25 females and 10 males), and 22 to 25 months (46 females and 15 males). Whenever possible, we used the same mice in multiple end points. A final sample size or end point can be found in the figure legends.

### Bulk RNA Sequencing and Data Analysis

The corneal epithelium from different ages (2, 12, 18, and 24–25 months) of B6 female mice was removed by debridement (*n* = 5/group; one sample equals corneal epithelium taken from both eyes of the same animal) and snap frozen in a tube containing RNA lysis buffer (Qiagen, Valencia, CA, USA). Total RNA was extracted using a QIAGEN RNeasy Plus Micro RNA isolation kit according to the manufacturer's instructions. The concentration was assessed using a NanoDrop 2000 (Thermo Fisher Scientific, Waltham, MA, USA).

A double-stranded DNA library was created at Baylor College of Medicine's Genomics and RNA Profiling Core. Briefly, using 100 ng of total RNA (measured by pico green), an oligodT primer containing an Illumina-compatible sequence at its 5′ end was hybridized to the RNA, and reverse transcription was performed using a Lexogen kit. Second-strand synthesis was initiated by a random primer containing an Illumina-compatible linker sequence at its 5′ end. The purified double-stranded library was then amplified and purified. The resulting libraries were quantified using Qubit 2.0 (Thermo Fisher Scientific) and fragment size was assessed with the Agilent Bioanalyzer (Agilent Technologies, Santa Clara, CA, USA). A quantitative PCR (qPCR) was performed on the libraries to determine the concentration of adapter-ligated fragments using Applied Biosystems ViiA7 Real-Time PCR System and a KAPA Library Quant Kit (Waltham, MA, USA). All samples were pooled equimolarly, requantified by qPCR, and reassessed on the bioanalyzer. Using the concentration from the ViiA7 qPCR machine above, 1.8 pM of the equimolarly pooled library was loaded onto NextSeq 500 high-output flow cell (Illumina, San Diego, CA, USA). PhiX Control v3 adapter-ligated library (Illumina) was spiked in at 1% by weight to ensure balanced diversity and to monitor clustering and sequencing performance. A single-read 75 base pair cycle run was used to sequence the flow cell. An average of 21 million reads per sample was sequenced. The FastQ file generation was executed using Illumina's cloud-based informatics platform, BaseSpace Sequencing Hub (Illumina).

Data were analyzed by ROSALIND (https://rosalind.bio/), with a HyperScale architecture developed by ROSALIND, Inc. (San Diego, CA, USA). Reads were trimmed using cutadapt^57^ and quality scores were assessed using FastQC. Reads were aligned to the Mus musculus genome build GRCm38 using STAR.^58^ Individual sample reads were quantified using HTseq^59^ and normalized via relative log expression using the DESeq2 R library.^60^ Read distribution percentages, violin plots, identity heatmaps, and sample multidimensional scaling plots were generated as part of the QC step using RSeQC.^61^ DEseq2 was also used to calculate fold changes and *P* values and perform optional covariate correction. The clustering of genes for the final heatmap of differentially expressed genes (DEG) was done using the partitioning around medoids method using the fpc R library. Hypergeometric distribution was used to analyze the enrichment of pathways, gene ontology, domain structure, and other ontologies. The topGO R library^62^, was used to determine local similarities and dependencies between Gene Ontology terms to perform Elim pruning correction. Several database sources were referenced for enrichment analysis, including Interpro,^63^ NCBI,^64^ MSigDB,^65^ REACTOME,^66^ and WikiPathways.^67^ Enrichment was calculated relative to a set of background genes relevant to the experiment. Venny 2.1.0 (Venny 2.1, BioinfoGP, https://bioinfogp.cnb.csic.es/tools/venny) was used to identify the common and overlapping genes between age groups and diagrams were generated. From the list of DEGs identified by RNA sequencing, we used the Metascape database (http://metascape.org/) to identify the altered enriched ontology clusters and biological pathways. To achieve this, we submitted the lists of DEGs and performed genome pathway enrichment analysis and Gene Ontology annotation.

### RNA Isolation and qPCR

The corneal epithelium of female and male B6 mice of two ages (3 months [8 females and 10 males) and 23–25 months [9 females and 10 males]) was harvested by debridement using a dulled blade and the tissue was placed in RNA lysis buffer and snap-frozen at −80°C until RNA extraction. Total RNA was extracted using a QIAGEN RNeasy Plus Micro RNA isolation kit (Qiagen). The RNA concentration was measured using a NanoDrop 2000 (Thermo Fisher Scientific). The cDNA was synthesized using the Ready-To-Go You-Prime First-Strand kit (GE Healthcare, Chicago, IL, USA). Real-time PCR was performed using specific TaqMan minor groove binder probes for small proline rich protein 1A (*Sprr1a*, Mm01962902_s1), IL-33 (*Il33*, Mm00505403_m1), unc-51 like autophagy activating kinase 1 (*Ulk1*, Mm00437238_m1), cyclin-dependent kinase inhibitor 1A (*Cdkn1a*, Mm04205640_g1), cyclin-dependent kinase inhibitor 2A (*Cdkn2a*, Mm00494449_m1), matrix metalloproteinase (MMP)-2 (*Mmp2*, Mm00439506_m1), aldehyde dehydrogenase 1 family member A1 (*Aldh1a1*, Mm00657317_m1), decorin (*Dcn*, Mm00514535_m1), collagen type I alpha 2 chain (*Col1a2*, Mm00483888_m1), collagen type VI alpha 1 chain (*Col6a1*, Mm00487160_m1), insulin-like growth factor binding protein 2 (*Igfbp2*, Mm00492632_m1), pigment epithelium-derived factor (*Serpinf1*, Mm00441270_m1), and TaqMan Universal PCR Master Mix AmpErase UNG in a commercial thermocycling system (StepOnePlus Real-Time PCR System Thermo Fisher Scientific), according to the manufacturer's recommendations.

The hypoxanthine phosphoribosyltransferase 1 (*Hprt1*; Mm00446968) gene was used as an endogenous reference for each reaction. The qPCR results were analyzed by the comparative Ct method and were normalized by the Ct value of *Hprt1*. The young group served as calibrators.

### Corneal Mechanical Sensitivity Measurement

Corneal sensitivity was measured with a Cochet-Bonnet esthesiometer (Luneau Ophthalmologie, Chartres Cedex, France) in unanesthetized naïve mice of both sexes at different ages: 2 to 3 months (13 females and 9 males), 12 months (20 female and 5 males), 18 months (10 females and 10 males), and 22 to 24 months (22 females and 6 males), as previously described.^54^ In brief, mice were restrained manually, and the central cornea was touched with the end of the esthesiometer's nylon filament. A positive response was recorded upon a blink reflex or a retraction into the ocular orbit. The longest filament length with a positive response (eyeblink) was measured as the corneal sensitivity (0 [min] to 6 [max]). Because corneal sensitivity can change depending on the time of the day,^68^ all measurements were taken at 10:30 am ± 20 minutes.

### Corneal Wound Epithelial Debridement and Evaluation of Wound Closure

Mice were subjected to unilateral epithelial debridement under general anesthesia using an isoflurane vaporizer (SomnoSuite, Kent Scientific Corporation, Torrington, CT, USA). A 2-µL drop of topical proparacaine was applied to the eye and blotted dry with filter paper. The central corneal epithelium was demarcated with a 1.5-mm trephine (McKesson Argent Surgical Systems, Richmond, VA, USA)^21^ followed by removal of the epithelium using a sterile disposable blunt blade under a dissecting microscope. Mice received an analgesic diet containing carprofen (2 mg/tablet, Bio-Serv, SMD 150-2, 1 tablet per mouse per day) 24 hours before the debridement until 48 hours after the procedure. No other eye drops (such as antibiotics) were used. Mice were routinely checked after surgery.

Wound closure was evaluated at different time points (1, 20, 24, 28, and 48 hours) after injury. The healing rate was calculated by measuring the re-epithelialization area as compared with the 1-hour time point area, which was set to 100%. Briefly, mice were anesthetized and 2 µL of 0.1% sterile sodium fluorescein solution in PBS was applied to the wounded eye followed by rinsing with 2 mL of balanced salt solution (Alcon Laboratories Inc, Fort Worth, TX, USA). The cornea was photographed under a LZM microscope (Nikon SMZ1500, Nikon, Japan) with an LED light source at an excitation of 488 nm (Lumencor Inc, Beaverton, OR, USA) using a digital camera (Zyla sCMOS, Andor Technologies, Belfast, UK). Image analysis was performed using NIS-Elements imaging software (Advanced Research version 5.30.04, Nikon). Wound size measurements were transferred to an Excel database, where the results were averaged within each group and analyzed.

### Evaluation of Corneal Barrier Function and Residual Haze 14 Days After Debridement

Corneal barrier function as the uptake of sodium fluorescein was evaluated in both eyes at days 14, 21, and 28 after injury after addition of 2 µL of 0.1% sodium fluorescein stain, rinsing with balanced salt solution, and imaging as described elsewhere in this article for assessing wound closure under brief anesthesia with isoflurane. Mice were euthanized at day 35 after injury with an overdose of isoflurane, followed by cervical dislocation, but no imaging was performed at this time point. Corneal opacity was investigated in bright field images captured using the LZM microscope with a color digital camera (Zyla sCMOS, Andor Technologies) and high-intensity illuminator (NI-150, Nikon). Corneal images were graded clinically as published.^69^ Injured and noninjured eyes were photographed. Eyes that had corneal opacity were counted and cumulative data were submitted for statistical analysis using the χ^2^ test. These eyes were also excluded from the evaluation of corneal barrier function on day 14 because they could interfere with the dye spreading on the cornea, but they were not excluded from the day 21- or 28-day analysis.

### Statistical Analysis

Two-way ANOVA or Kruskal–Wallis nonparametric tests followed by multicomparison tests were used for statistical comparisons of corneal sensitivity by sex, wound size biological replicates, fluorescein staining mean intensities, and gene expression analysis. Pearson correlation was used to correlate age with corneal sensitivity. The Mann–Whitney *U* test was used for same-sex age comparisons of corneal sensitivity and the χ^2^ test was used to analyze corneal opacity on follow-up. A *P* value of 0.05 or less was considered statistically significant. These tests were performed using GraphPad Prism 9.4.1 software (GraphPad Incorporation, San Diego, CA, USA). After completion of all experiments, data were averaged and graphs were generated. The final sample per experiment is shown in the figures and graphs.

## Results

### Gene Expression Analysis in the Female Corneal Epithelium Identifies Several DEGs With Age

Aging is a significant risk factor for dry eye disease.^70^ We first investigated the alterations in molecular pathways in the corneal epithelium in naïve female B6 mice of different ages (2, 12–13, 18, and 24 months). We collected corneal epithelium, extracted RNA and performed bulk RNA sequencing. Data was analyzed using ROSALIND software with the parameters of at least a 1.2-fold change and a false discovery rate of 0.05 or less.

**Figure 1.**
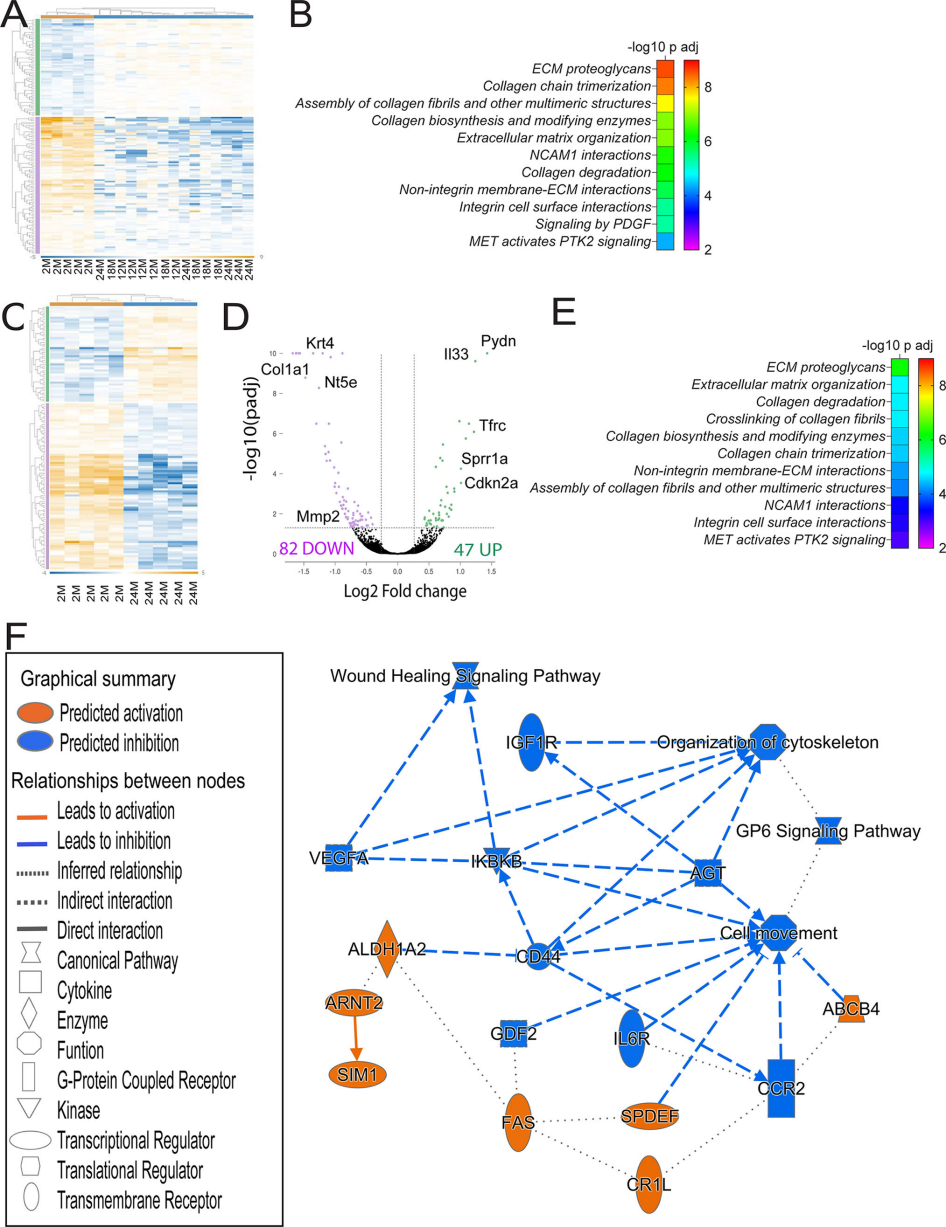
Transcriptome changes in the corneal epithelium of aging mice. (**A**) Overall heat map profile for 157 DEGs in the corneal epithelium comparing young corneal epithelium (*left*) to corneal epithelium of different ages (12–24 months). (**B**) Relative enrichment based on pathway analysis generated with Qiagen IPA comparing young to aged corneas irrespectively of age (12- to 25-month range). (**C**)**.** overall heat map profile for 129 DEGs in the corneal epithelium comparing young (left) to 24- to 25-month corneal epithelium. (**D**) A volcano plot showing the differences in expression and the magnitude of change in the corneal epithelium modulated by aging between the young (2 months) and aged (24–25 months) corneas. 82 genes were down-regulated and 47 were up-regulated. Each dot represents one gene. The dotted line indicates a *P* value of 0.05. (**E**) Relative enrichment based on pathway analysis generated with Qiagen IPA comparing young to 24- to 25-month corneas. (**F**) Graphical abstract generated by IPA comparing young to 24- to 25-month corneas.

Initially, we compared the young group to all aged groups. Unsupervised clustering demonstrated that young corneal samples clustered separately from the older samples, regardless of age (Fig. 1A). Among the aged samples, the 12-month group had four of the five samples clustered together, while there was variability among the 18- and 24-month samples, as expected with aged specimens. There were 157 DEGs (65 upregulated and 92 downregulated) (Supplementary File 1). ROSALIND/Reactome analysis of pathways showed that these DEGs have been identified in several pathways: extracellular matrix proteoglycans; collagen trimerization, assembly, collagen biosynthesis, and degradation; and extracellular matrix organization. Additional pathways are neural cell adhesion molecule 1 interactions, nonintegrin membrane–extracellular matrix interactions, signaling by platelet-derived growth factor, and mesenchymal–epithelial transition activation (Fig. 1B). Further predicted pathways can be found in Supplementary File 2.

The number of DEGs compared with young (2 months old) increased with increased age (64, 94, and 129 DEGs) (Supplementary File 3). We followed up by investigating the DEGs that are common or unique to each age comparison using Venn diagrams, regardless of their expression (upregulated or downregulated) (Supplementary Fig. S1 and Supplementary File 4). Only 10 DEGs were common between 12- to 13-month-old vs. young and 18-month-old vs. young comparisons, whereas 29 DEGs (15.7%) were common between all three of the comparisons. There were 66 DEGs that were unique to the 24-month vs. 2-month comparison (Supplementary Fig. S1A).

Because the greatest number of DEGs was observed between the youngest group (2 months old) vs. the oldest (24 months old), we performed a more in-depth investigation of this comparison (Figs. 1C–F). A volcano analysis showed that, among the 129 DEGs, 82 were downregulated and 47 were upregulated (Fig. 1D). The most upregulated gene in the aged group was *Pydn* (prodynorphin, 1.43-log2 fold change), followed by *Il33* (1.2-log2 fold change), whereas the most downregulated gene was *Wfdc18* (−1.67 log2-fold change). ROSALIND/Reactome analysis in this comparison showed that many pathways that were highlighted in Fig. 1B (comparing young to all age groups) were still significant when comparing the 24-month-old group with the young group, albeit the adjusted *P* value was smaller (Fig. 1E, Supplementary File 5). We also performed a meta-analysis of the combined three aged groups vs. the young group using ROSALIND (Supplementary Fig. S2). Using this tool, 73 genes were upregulated with aging and 113 genes were downregulated. The top five upregulated biological pathways involve regulation of protein stability, regulation of protein kinase activity, hormone metabolic process, cellular response to drug, and negative regulation of protein phosphorylation. The top five downregulated biological pathways involved negative regulation of hydrolase activity and peptidase activity, sensory perception, blood vessel development, and wound healing (Supplementary Fig. S2).

Next, we uploaded the list of the DEGs into IPA (Qiagen) and we performed canonical analysis. The pathways with a negative Z-score (indicating predicted downregulation) are GP6 signaling, wound healing signaling, pulmonary fibrosis idiopathic signaling, dendritic cell maturation and others (Supplementary File 6). Interestingly, many of the DEGs involved in these pathways are genes that encode for different subsets of collagen, such as *Col1a1*, *Col1a2*, *Col4a1*, *Col5a1*, *Col5a2*, *Col6a1*, and *Mmp2*.

The graphical abstract by IPA explores and predicts the relationship among all the DEGs and it is shown in Fig. 1F. As previously identified by the canonical pathway analysis, wound healing signaling pathway is predicted to be decreased, as well as organization of cytoskeleton and cell movement, and insulin-like growth factor 1 receptor. The kinase inhibitor of nuclear factor kappa-B kinase subunit beta is also predicted to be decreased, as well as CD44 and IL-6R. CD44 is a glycoprotein that is important for epithelial and immune cells and is also a receptor for hyaluronan.^71^ It is predicted that Aldh1A2 (an enzyme that catalyzes the synthesis of retinoic acid from retinaldehyde), Fas (a death receptor involved in apoptosis), and the adenosine triphosphate-binding cassette 4 are upregulated.

**Figure 2.**
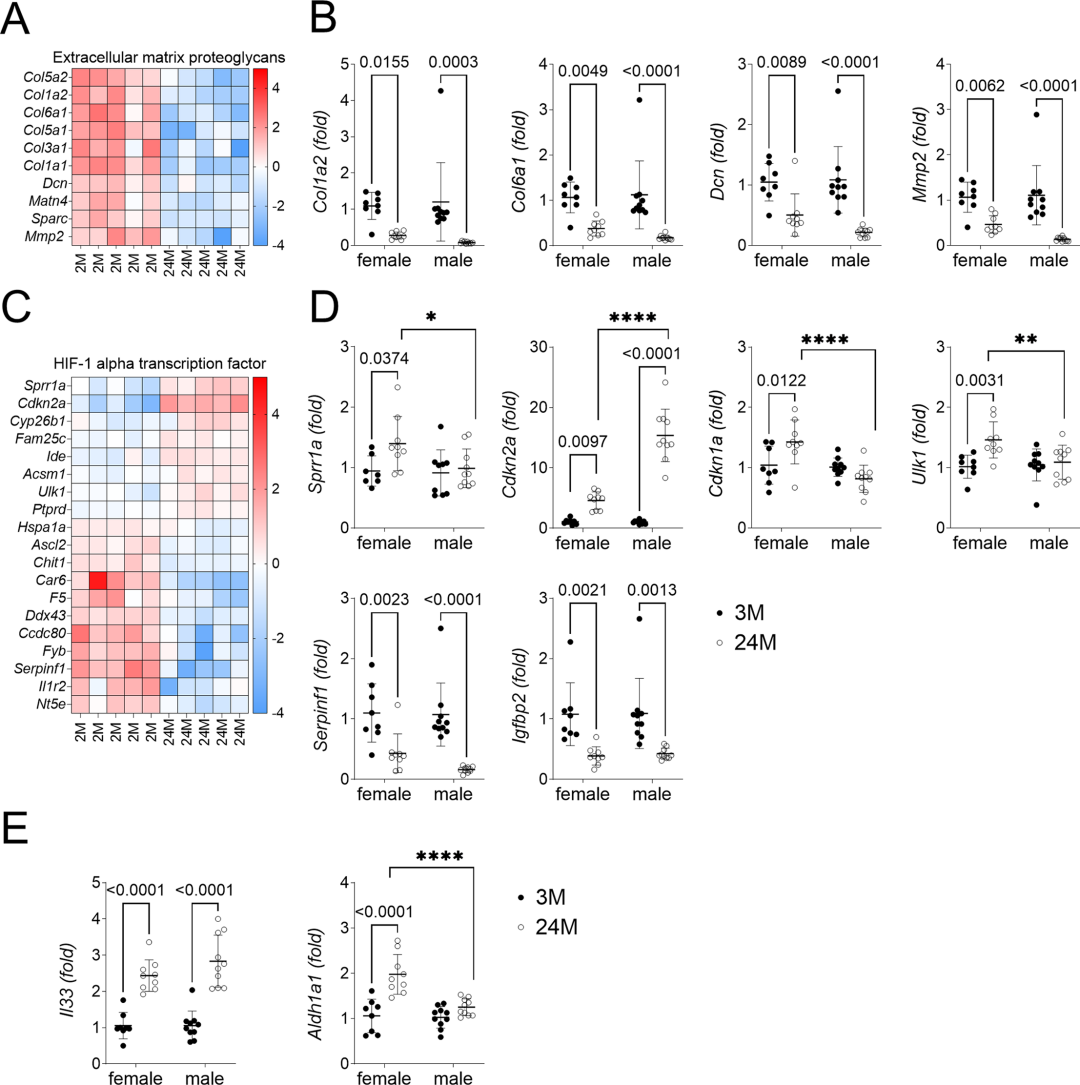
Aged corneal epithelium shows altered transcriptome. Corneal epithelium from female mice was scraped and subjected to bulk RNA sequencing. Validation using individual qPCR assays was performed in additional female corneas not subjected to bulk RNA sequencing and the effect of aging was compared male corneas. (**A**) Heatmaps showing DEGs related to extracellular matrix proteoglycans in female corneas. (**B**) Relative fold of expression of *Col1a2*, *Col16a1*, *Dcn*, and *Mmp2* in male and female corneas. (**C**) Heatmaps showing DEGs related to the transcription factor HIF-1α pathway in female corneas. (**D**) Relative fold of expression *of Sprr1a*, *Cdkn2a*, *Cdkn1a*, *Ulk1*, *Serpfin1*, and *Igfbp2* in male and female corneas. (**E**) Relative fold of expression *of Il33*, and *Aldh1a1* in male and female corneas. (**A** and **C**) Data was analyzed using ROSALIND software with the parameters fold change ≥1.2-fold or ≤1.2-fold change and a false discovery rate (FDR) ≤ 0.05. (**B**, **D**, and **E)** Two-way ANOVA with Sidak's comparison test*. P* value as shown or as *P < 0.05; **P < 0.01; ****P < 0.0001. Each dot represents one biological sample (both corneas pooled from the same animal, *n* = 8–10/sex/age).

Next, we chose two differentially expressed pathways identified by bulk RNA sequencing and validated using individual PCR reactions. For this validation, we included samples from both female and male corneal epithelium. Fig. 2A shows detailed heatmaps of genes involved in the extracellular matrix proteoglycans. There was decreased expression of multiple extracellular matrix proteoglycans with age, including *Col5a2*, *Col1a2*, *Col6a1*, *Col5a1*, *Col3a1*, *Dcn*, *Sparc*, and *Mmp2*. Male samples showed a similar decrease in *Col1a2*, *Col6a1*, *Dcn*, and *Mmp2* in aged corneas (Fig. 2B).

The second pathway we validated with qPCR was the hypoxia-inducible factor (HIF-1α) transcriptional factor associated pathway (Fig. 2C). Confirming our bulk RNA sequencing results, aged female corneas showed an upregulation of *Sprr1a*, *Cdkn2a*, and *Ulk1*, and a downregulation of *Serpinf1.* Interestingly, in aged male corneas we did not observe upregulation of *Sprr1a*, *Cdkn1a*, or *Ulk1*. In contrast, the increase in *Cdkn2a* in male corneas was significantly higher than in the aged female group (Fig. 2D)*. Sprr1a encodes* small proline-rich protein *1A* (SPRR1A), which is expressed in squamous epithelia such as in skin*.* Upregulation of SPRR1A has been linked to corneal metaplasia in dry eye disease.^72^ *Cdkn2a* and *Cdkn1a* encode p16 and p21, respectively, proteins involved in cell senescence.^73^ An increase in *Cdkn2a* has been reported in aged corneal epithelium*.*^55^ *Serpinf1* encodes pigment epithelium-derived factor (PEDF), which has anti-angiogenic and anti-inflammatory properties.^74^^–^^76^ *Igfbp2* (encoding insulin-like growth factor binding protein 2), a downstream gene in the HIF-1α pathway,^77^ was also decreased in aged female and male corneas. Finally, we chose to empirically validate *Il33*, linked to ocular inflammation,^78^ and *Aldh1a1*, which is modulated by aging.^56^ *Il33* increased in both female and male corneas while *Aldh1a1* only increased in female mice (Fig. 2E). Taken together, these results indicate that age and sex impact the corneal epithelium transcriptome.

### Aged Mice Have Decreased Corneal Sensitivity and Sex-specific Differences

**Figure 3.**
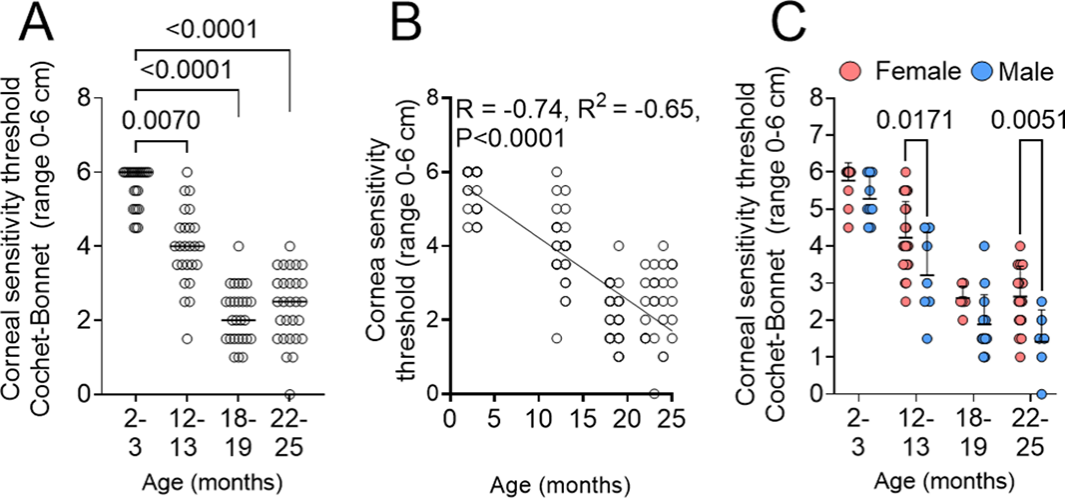
Age- and sex-specific differences in corneal mechanical sensitivity. (**A**) Comparison of corneal mechanical sensitivity measured with the Cochet Bonnet esthesiometer in mice of different ages. Nonparametric Kruskal–Wallis test followed by Dunn's comparison test. *P* values as shown. (**B**) Pearson's correlation of cornea mechanical sensitivity and age. R^2^ = coefficient of determination; R = coefficient of correlation. (**C**) Comparison of corneal mechanical sensitivity measured with the Cochet Bonnet esthesiometer in mice of different ages split by sex. Nonparametric two-way ANOVA with Sidak's multicomparison test. *P* values as shown.

Corneal epithelial cells and corneal nerves have a symbiotic relationship.^21^^,^^79^ We have published that 24-month-old female B6 mice have decreased corneal nerve density and corneal sensitivity,^41^ whereas others have also noted a decrease in cornea sensitivity with aging.^80^^,^^81^ Here we confirm and extend our previous studies by assessing corneal sensitivity in multiple ages of both female and male mice (2–3, 12–13, 18–19, and 22–25 months). Corneal mechanosensitivity was measured using the Cochet–Bonnet esthesiometer in mice of different ages and values were plotted. We observed that corneal mechanosensitivity progressively decreased with increasing age until a plateau was reached around 18 to 19 months of age (Fig. 3A). Pearson's correlation analysis showed an inverse correlation of cornea sensitivity and age (R = −0.0815; R^2^ = −0.65; *P* < 0.0001) (Fig. 3B). When the data were segregated by sex, we observed that male mice aged 12 to 13 months and 22 to 25 months had lower corneal sensitivity than female mice of the same age (Fig. 3C).

### Aged Mice Have a Delayed Corneal Re-epithelialization Rate After Corneal Wound Debridement

**Figure 4.**
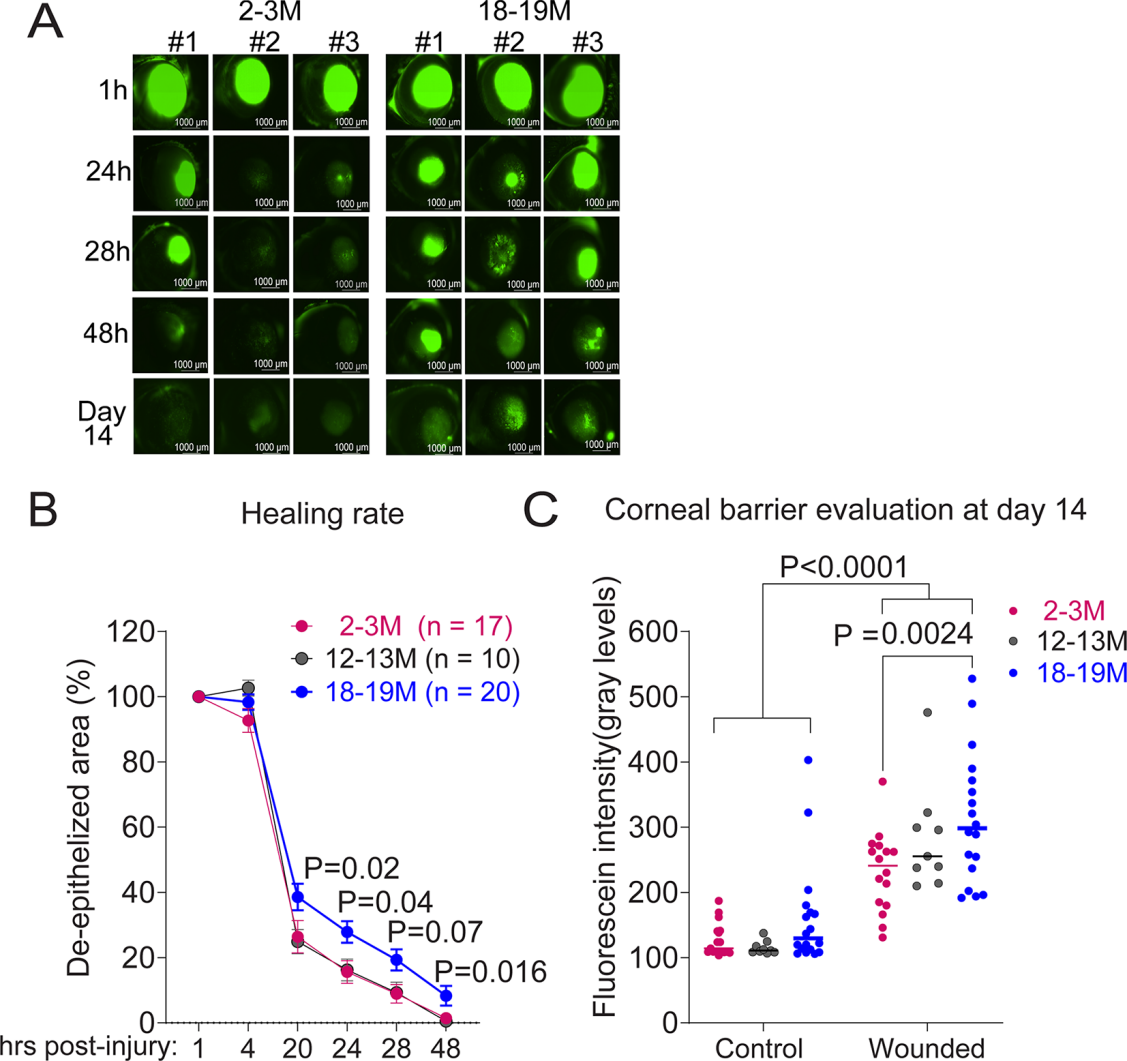
Aged corneas heal more slowly than young after corneal epithelial debridement. A unilateral corneal epithelial debridement wound was created in mice under anesthesia as described in the methods. To measure the de-epithelialized area, 0.1% fluorescein was topically applied followed by washing. Corneas were photographed under the LZM microscope and analyzed using image analysis software. The size of the epithelial wound at 1 hour was set as 100%. Mice of both sexes were used. (**A**) Representative images of corneas stained with 0.1% fluorescein after debridement at different time points (1, 24, 28, and 48 hours) (**B**) Rates of re-epithelialization in the experimental groups. Corneal re-epithelialization rates were decreased in both aged male and female mice in comparison to the young mice. Two-way paired ANOVA with Dunnet's multicomparison test. *n* = 10–20/group (2–3 months, *n* = 17; 12–13 months, *n* = 10; 18 months, *n* = 20). Overall significance of *P* < 0.0001; *P* values of individual time points as shown. (**C**) Corneal barrier evaluation at day 14 debridement. Cumulative data of corneas stained with 0.1% sodium fluorescein 14 days after injury. Each dot represents one eye separated into control (no injury) and wounded. Two-way ANOVA with Sidak's comparison test. *P* value as shown.

In our previous publication, a decrease in corneal sensitivity in 24-month-old mice was accompanied by a decrease in corneal axon density.^41^^,^^82^ Because corneal nerves play a crucial role in the wound healing process of the corneal epithelium, we hypothesized aged corneas would heal more slowly than young corneas. To investigate this, we performed epithelial corneal debridement in mice of different ages (2–3, 12–13, and 18–19 months) using a dull blade. Pilot studies using our protocol show that the basement membrane was intact 1 hour after the initial wound (data not shown). Our results are summarized in Figures 4A, 4B. Because 24-month-old mice are more fragile and difficult to obtain, we did not attempt to use this age group. Initially, we separated mice based on age and sex, but because we did not observe a sex-specific effect, data are aggregated by age only. We observed that 18- to 19-month-old mice heal more slowly compared with 2- to 3-month-old mice and 12- to 13-month-old mice. Interestingly, the 12- to 13-month-old mice healed as fast as the young group (Figs. 4A, 4B).

The greatest differences in re-epithelialization rate in the 18- to 19-month-old mice group were observed at 20, 24, and 48 hours after debridement. There was still a significant number of unhealed corneas in the 18- to 19-month-old group at the 48-hour mark (*P* = 0.04) (Supplementary Fig. S3A). These results indicate that aging impacts the initial corneal wound re-epithelialization rate.

### Aged Mice Had Worse Corneal Barrier Function on Day 14 After Debridement

To investigate if normal corneal barrier function was restored after re-epithelialization, we assessed corneal permeability to sodium fluorescein 14 days after debridement, when the corneal epithelial wound was expected to have closed (Supplementary Fig. S3B). Corneal fluorescein staining was higher at all ages and in both sexes of mice subjected to debridement wounding after 14 days. However, corneal fluorescein staining in the 18-to 19-month-old mice was significantly higher than those seen in the 2- to 3-month-old mice (Figs. 4A, 4C), suggesting increased corneal permeability and epithelial barrier disruption with age. These results indicate that aging affects the recovery of the corneal epithelium after injury, which could contribute to corneal epithelial barrier distribution seen in age-related dry eye disease.

### Aged Mice Are More Likely to Develop Epithelial Erosions After Debridement Compared With Young Mice

**Figure 5.**
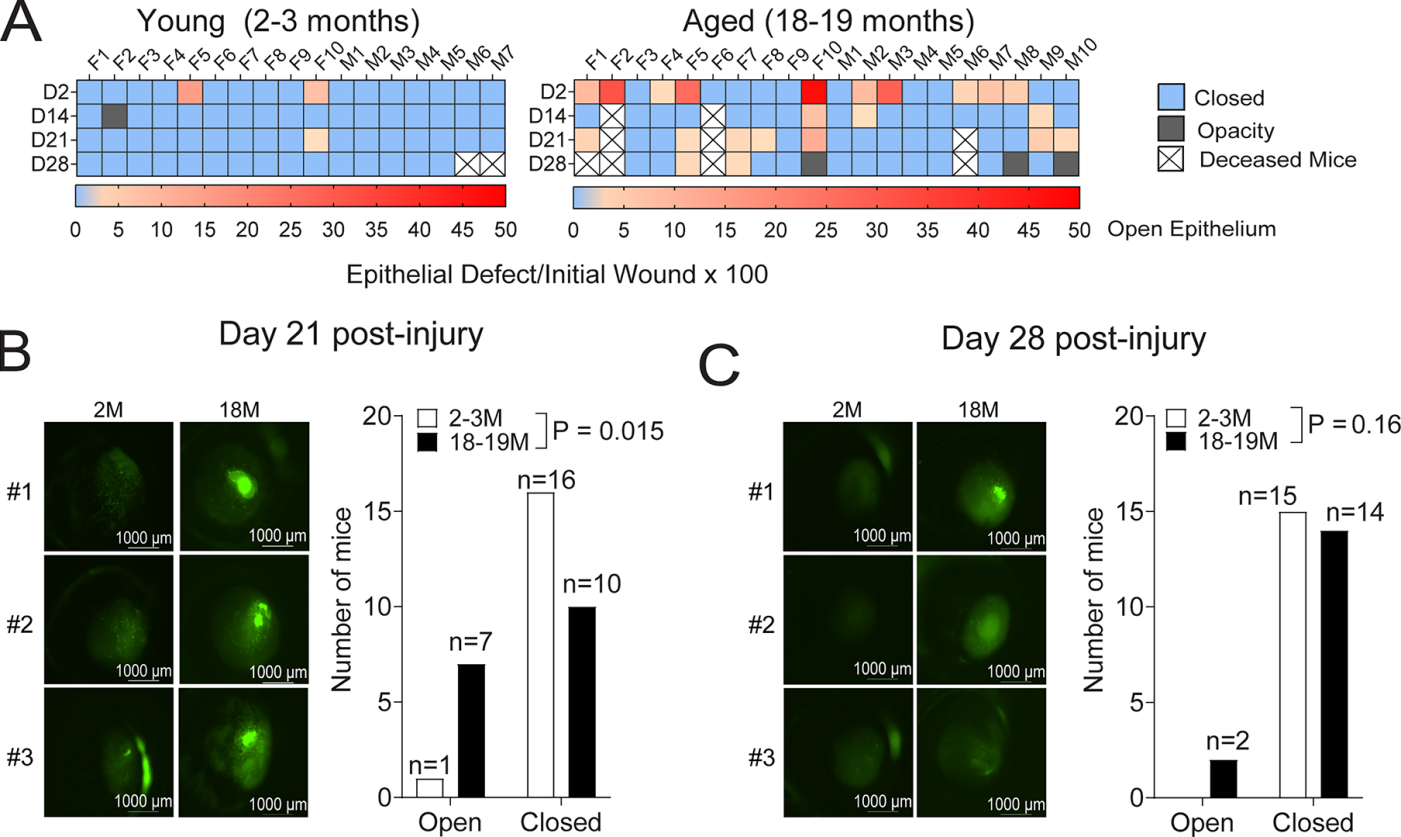
Aged mice develop epithelial erosions. Mice were followed up after 21- or 28-days after injury and the wound size was measured as described in the methods. Corneas were classified categorically as open or closed depending on if epithelial erosions were present or not (irrespectively of the size). (**A**) Time course of epithelial erosions in young mice (2–3 months) and aged (18–19 months) at different time points after debridement (48 hours, day 14, day 21, and day 28) showing individual mice. At all time points, the de-epithelialized area was measured and compared with the area at the 1-hour time point after wounding to calculate the re-epithelialization rate. Values were plotted from 0 (close wound/no erosion) to 50 (maximum value in both young and aged). Grey box indicates opacity formation, X box indicates mouse deceased. F, female; M, male. (**B**) Representative images of fluorescein-stained corneas at day 21 after debridement and cumulative data of incidence of epithelial erosions in young and aged group. χ^2^ test. (**C**) Representative images of fluorescein-stained corneas at day 28 after debridement and cumulative data of incidence of epithelial erosions in young and aged group. χ^2^ test.

Because there was no difference in wound healing and barrier function between the young and the 12- to 13-month-old group, we opted to continue our studies by comparing only the 2- to 3-month-old with the 18- to 19-month-old group for erosion formation. We investigated the appearance of epithelial erosions at day 21 and at day 28 after injury. All 2- to 3-month-old mice survived until day 21, but two died before the day 28 time point, reducing the sample size from 17 to 15 (Fig. 5A). In the 18- to 19-month-old group, two mice died before day 14, one died before day 21, and one died before day 28 after debridement, reducing the sample size from the initial 20 mice to 16 by the day 28 time point (Fig. 5A). These deaths did not seem to be related to the corneal debridement procedure; unexpected death in aged mice is a frequent event.

All surviving mice received topical fluorescein and the presence or absence of de-epithelialized corneas was then counted and plotted using the χ^2^ test to investigate if the frequency was significant (Figs. 5B, 5C). On day 21, we identified an increased incidence of epithelial erosions in the 18- to 19-month-old group (7/17 mice) (Figs. 5A, 5B). There were no sex differences among the mice that had epithelial erosions (Fig. 5A). A further follow up at day 28 after debridement showed that 2 of the 16 aged corneas still had epithelial erosions, but this difference was not statistically significant (*P* = 0.16). Although aged mice had more corneal opacifications than young mice (3 vs. none), this was not statistically significant either (data not shown). These results indicate that aged mice not only show a delayed rate of re-epithelialization in the first 48 hours, but also have an increased number of epithelial erosions after debridement beginning at day 14 and lasting through day 28 after injury.

## Discussion

Aging is a complex biological process that is accompanied by a slow decrease in function in many tissues. The eye is no different. The cornea needs to maintain clarity to keep its refractory power intact and to maintain ocular comfort. In our study, we described how aged corneas have an altered transcriptome and the consequent ability for the corneal epithelium to heal after debridement. We identified an increased number of DEGs in the corneal epithelium with increasing age. The majority of these DEGs were related to collagen assembly/machinery/alignment and extracellular matrix deposition. This finding was true when comparing young corneal epithelium with any epithelium older than 12 months of age.

Our studies in naïve female corneas using bulk RNA sequencing and qPCR validation using female and male samples showed a decrease in genes involved in extracellular matrix proteoglycans. Collagen fibril organization in the stroma is essential for cornea clarity. A previous study showed that aging decreases tissue remodeling by MMPs and decreases the structural integrity of collagen fibers,^83^ possibly suggesting that with age comes a diminished capacity for the corneal epithelium to remodel collagen as needed to maintain clarity. Another important pathway identified in our study is extracellular matrix deposition, which is essential in cell signaling, cell migration, and maintenance of the limbal stem cell niche.^84^^,^^85^ Extracellular matrix deposition and function have long been known to be altered with advanced age. Age is associated with decreased expression of genes that regulate the circadian clock.^86^^–^^88^ The cell signaling pathways regulated by circadian rhythms become increasingly dysregulated with advancing age and these pathways include genes that regulate both extracellular matrix gene expression and its assembly.^87^^,^^89^^–^^91^

Our analysis using IPA, which predicts which pathways are altered between different transcriptomes, identified several pathways likely to be influenced by collagens. These included cell migration, organization of cytoskeleton, and wound healing. The meta-analysis using ROSALIND identified HIF-1α as a key transcription factor altered in aged corneas. Previous studies have shown that HIF-1α is altered during aging^92^^,^^93^ and a deficiency of HIF-1α in skin epidermis increased epidermal aging and altered re-epithelialization in both humans and mice.^93^^,^^94^ HIF-1α knockdown suppressed HIF-1α–dependent claudin-1 expression and epithelial barrier function,^95^ and a study using a mouse model of closed eye contact lens wear suggested that HIF-1α was a proximal regulator of vascular endothelial growth factor expression in the corneal epithelium.^93^ Because HIF-1α has been given an essential role in wound healing,^96^^,^^97^ investigating the HIF-1α signaling pathway may open novel enquiries for future studies in age-related corneal epithelium diseases. Our gene expression analysis also showed a decrease in *Serpinf1* (encoding PEDF), a downstream gene in the HIF-1α pathway, which was downregulated in both male and female corneas. PEDF expression has been found in all layers of the corneal epithelium.^75^^,^^76^^,^^98^ Peptides derived from PEDF bind to collagen and the extracellular matrix ^99^^,^^100^ and have been hypothesized to work as a sponge to localize the anti-angiogenic protein in the corneal stroma. A 44-amino acid peptide derived from PEDF plus docosahexaenoic acid, an omega-3 fatty acid, together were found to promote nerve regeneration.^74^ PEDF has also been used as a potential new therapy for dry eye disease. Recombinant PEDF ameliorated dry eye signs while anti-PEDF worsened dry eye signs in mice.^75^

Our results from the bulk RNA sequencing and individual validation of DEGs showed that *Aldh1a1* was upregulated in the corneal epithelium of the 24M corneas. A recent study demonstrated *Aldh1a1* is downregulated in aged murine corneas, and this was accompanied by a downregulation of canonical nuclear factor κB signaling.^56^ Discrepancies in the two studies could be related to the sex of the animals (both sexes [ours] vs. undefined^56^) and the stage of cornea disease. We isolated epithelial cell mRNA only from mice whose corneas had no visible macroscopic changes such as scars or opacifications while the previous studies described a 30% prevalence of corneal opacifications in aged mice.^56^

Corneal epithelial wound healing is an essential process that maintains the integrity and transparency of the cornea. It is dynamic and complex and involves cell migration, cell proliferation, and expression of numerous cytokines and chemokines by resident and recruited cells. As individuals age, they are subjected to environmental challenges that can alter crucial cell:cell and cell:matrix interactions.^101^^–^^103^ Cataracts, glaucoma, macular degeneration, and dry eye disease all increase in frequency with age and impair vision. A common feature shared by these pathologies is an age-related increase in inflammation.^104^^–^^107^ Nerves play a crucial role in both corneal homeostasis and in the wound healing process by maintaining epithelial integrity, providing sensory feedback, and releasing neurotransmitters and peptides that influence cellular responses.^108^^–^^112^ Our earlier publication showed that aging is associated with a decrease in intraepithelial corneal nerve density in B6 mice, and we identified a decrease in specific genes involved in encoding proteins that mediate axon growth and targeting.^41^ Here we confirmed that aged male and female mice have decreased corneal sensitivity when compared with young mice extending earlier reports.^41^^,^^113^ We also investigated sex differences and found middle aged (12–13 months of age) and old (22–24 months of age) female mice have higher corneal sensitivity than males of the same age. There was no significant sex difference observed at the 18-month timepoint. The existence of a sex-specific difference in corneal sensitivity is under some debate: a few previous studies in humans observed no difference between biological sexes^114^^–^^117^; however, one study in humans found that corneal sensitivity was greater in women and also reported an age-related decrease in corneal sensitivity, as we did here.^118^ One possible reason for these differences may be where on the cornea sensitivity is measured; one study found specific sex differences in human corneas only in the superior, temporal, and inferior areas of the cornea, but found no sex differences in sensitivity in the central or nasal areas.^119^

Epithelial and stromal alterations, including corneal opacification of unknown origin, have been described in naïve aged mice and dogs.^56^^,^^120^ For the past several years, we have maintained a colony of aged B6 mice and have observed corneal opacification in approximately 10% to 15% of naïve mice older than 18 months of age. Although we have observed corneal opacification in 12-month-old mice, it is less frequent. Interestingly, fluorescein staining remained elevated in 2-month-old mice after debridement, despite the fact that there were no erosions present. However, fluorescein staining in 22- to 24-month-old mice 14 days after injury was significantly greater than that seen in the 2-month-old mice. Together these data indicate that full corneal epithelial barrier reformation after injury takes longer than 14 days in mice of all ages. Furthermore, the increased incidence of erosion formation in older mice likely contributes to the increased fluorescein staining seen in the older mice.

We next investigated the effect of aging on the corneal epithelial wound healing response. To do this, we evaluated wounded corneas at six time points within the first 48 hours after corneal epithelial debridement. We observed significantly lower healing rates in the 18-month-old mice compared with the young group. Previous studies showed that 27-week-old B6 mice cornea wounds healed more slowly than 9-week-old B6 mice corneas over the first 24 hours.^121^ Likewise, in humans, corneal epithelial wound healing is delayed in aged individuals compared with younger people, possibly owing to a decrease in the regenerative capacity of the corneal epithelial cells.^122^ Another possible reason is that aging corneal epithelial cells may exhibit changes in their cellular response to injury or inflammation that can lead to prolonged inflammation and, consequently, delayed healing.^123^ Delayed corneal epithelium wound healing could also be associated with changes in the ocular surface tear film composition. An altered tear film can increase susceptibility to dry eye disease and other ocular surface conditions, all of which can negatively affect corneal epithelial health, creating a vicious cycle of inflammation and disease that may lead to delayed wound healing.^124^

Our results also show a higher incidence of epithelial defects after injury in aged mice compared with young mice. Persistent corneal epithelial defects, also referred to as corneal erosions, occur when the adhesion of the epithelium to the basement membrane is inadequate. Hemidesmosomes insert deeply into the basement membrane to maintain epithelial adhesion during homeostasis. To migrate in response to injury, hemidesmosomes disassemble and cells detach from basement membrane laminin and collagen VII. As the cells migrate as a sheet, MMPs secreted by the epithelial cells degrade the underlying basement membrane extracellular matrix. After re-epithelialization is complete, hemidesmosomes reassemble with the extracellular domains of cell adhesion proteins binding to basement membrane proteins secreted primarily by the corneal epithelial basal cells; if basement membrane synthesis or its reassembly is delayed or defective, then hemidesmosomes will fail to stabilize and erosions will form. The transcriptomic data presented here are obtained from unwounded aged female mice and are consistent with a decrease in the synthesis and secretion of matrix proteins by corneal epithelial cells with advancing age.

It has been challenging to understand the exact mechanisms that lead to recurrent corneal erosions. In a mouse model used to study erosion formation,^125^^,^^126^ naïve corneas with preexisting stromal scars were found to have a decreased frequency of erosions after debridement. In addition, removal of the basement membrane at the time of injury using a rotating burr also decreases the frequency of erosions. Patches of debris can become trapped beneath the corneal epithelial sheet during and after migration, delaying basement membrane reassembly, inducing MMP synthesis by resident immune and epithelial cells, and leading to senescence and apoptosis of overlying corneal epithelial cells. MMPs including MMP-9 degrade the extracellular domain of the a6b4 hemidesmosomal integrin decreasing cell:matrix adhesion and allowing erosions to form. The impact of aging on spontaneous corneal erosion formation in response to prior mechanical corneal debridement had not been reported previously.

Recurrent corneal erosions or persistent epithelial defects are names for a clinical syndrome that typically affects adult patients between 20 and 80 years of age, but the mean age of occurrence is between 40 and 50 years of age.^116^^,^^127^^–^^129^ Surgical treatments in the clinic include anterior stromal puncture and rotating burr polishing of the area around the erosion site.^130^^,^^131^ Anterior stromal puncture forces corneal epithelial cells to migrate into and out of puncture sites, increasing the surface area for adhesion and exposing them to stromal collagens, proteoglycans, and growth factors as the cell sheet migrates over the exposed wound bed. Rotating burr polishing removes debris and exposes the corneal epithelial cells to collagens and fibronectin that the integrins expressed by migrating epithelial cells adhere to. Both interventions lead to improved epithelial cell matrix adhesion. Although no sex differences were observed associated with the epithelial defects in our study, a retrospective study showed that women of the same age, with the same risk factors and treatment plans, healed significantly more slowly than men.^132^^,^^133^ A literature review reported that sex is not a risk factor for epithelial defects in dogs.^134^ This finding suggests that there may be species-specific differences in corneal healing, and more studies are needed to delineate the effect of sex on corneal epithelial defects.

Recurrent epithelial defects form for a variety of reasons, including basement membrane dystrophy, stem cell deficiency, viral infections, systemic diseases, or decreased nerve density that may inhibit the coordination of the healing response.^129^^,^^135^ Limbal stem cells are essential to maintain corneal epithelial homeostasis.^1^ This dysfunction of limbal stem cells can result in the loss of the corneal barrier and less corneal epithelial cell regeneration and healing.^136^^,^^137^ It has been shown that tissue regeneration ability decreases as stem cells change with aging.^138^ In patients with limbal stem cell deficiency, the most frequent causes were chemical or thermal injury to the ocular surface, Stevens–Johnsons syndrome (an autoimmune disorder), and congenital aniridia.^139^^,^^140^ Although it is possible that the delayed re-epithelialization of the cornea is caused by a defect in corneal stem cells, we do not believe that is the case in this study, because we did not observe decreases in the expression of mRNAs for corneal keratin Krt12 or an increase in expression of the conjunctival keratins (Krt8, Krt13, or Krt19) upregulated in stem cell deficiency.^141^^–^^145^

The data from our transcriptomics IPA pathway analysis suggest that matrix synthesis and assembly and cell migration pathways are downregulated with aging. Increased erosion formation in older mice could be mediated in part or entirely by defective reassembly of the epithelial basement membrane. Determining whether poor matrix synthesis and/or reassembly, aging stem cells, or inflammaging contribute most to the age-related changes we observe in corneal wound healing is beyond the scope of this work, and further investigation is necessary.

We have shown that aged corneas have an altered transcriptome and injuries to the corneal epithelium do not heal at the same rate as young corneas, without preference for sex. The corneal epithelium is constantly subjected to injuries caused by dust, pollen, eye rubbing, and blinking that cause transient loss of the corneal barrier that prevents infection and maintains corneal hydration. Our results increase our knowledge of the aging process by showing that age compromises the ability of the corneal barrier to be restored fully in response to injury and contributes to the development of epithelial erosions.

## Supplementary Material

Supplement 1

Supplement 2

Supplement 3

Supplement 4

Supplement 5

Supplement 6

Supplement 7
