# Supplementary material for: Age-Related Differences in the Mouse Corneal Epithelial Transcriptome and Their Impact on Corneal Wound Healing
Source: Invest Ophthalmol Vis Sci. 2024 May 13;65(5):21. doi: 10.1167/iovs.65.5.21 (PMC11098051; doi:10.1167/iovs.65.5.21)
Supplement: Supplement 1 [file iovs-65-5-21_s001.pdf]

Supplemental Figures

Supplemental Figure 1

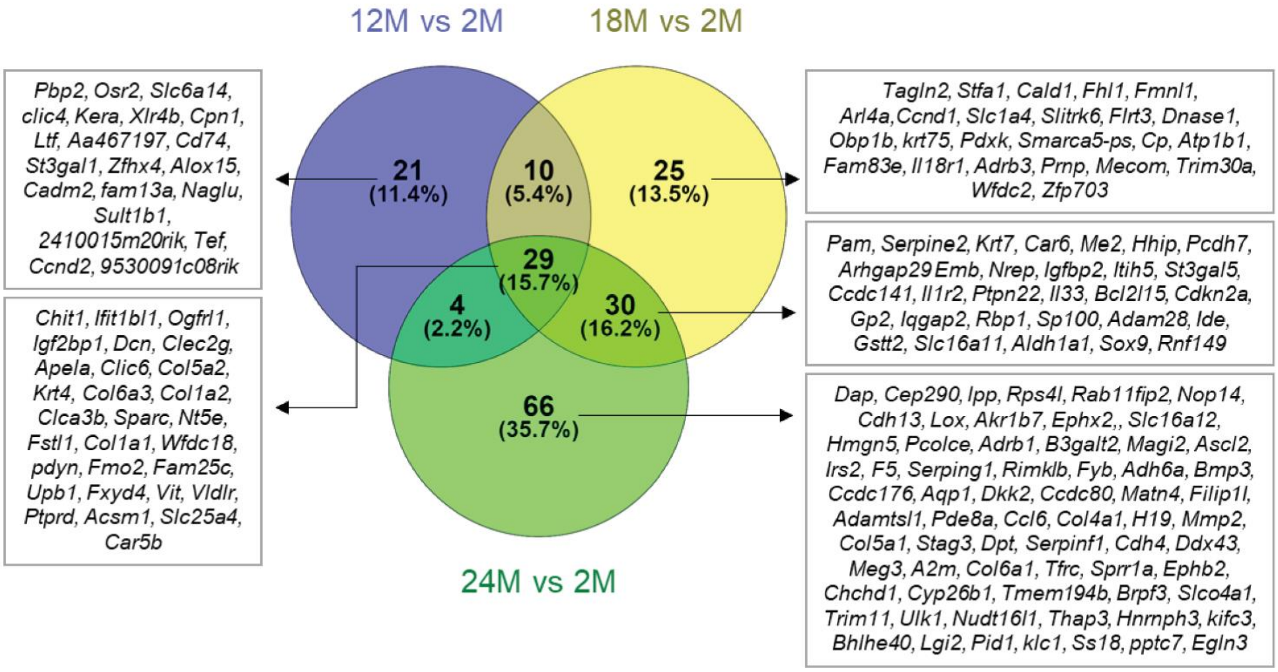

Supplemental Fig. 1. Analysis of the DEGs across all aged groups show aged mice have altered biological pathways. Corneal epithelium from all age groups were scraped, total RNA extracted and submitted for bulk RNA sequencing. DEGs were analyzed using ROSALIND. Venn diagrams listing down- and up-regulated DEGs between age groups, including the common genes among them.

Supplemental Figure 2

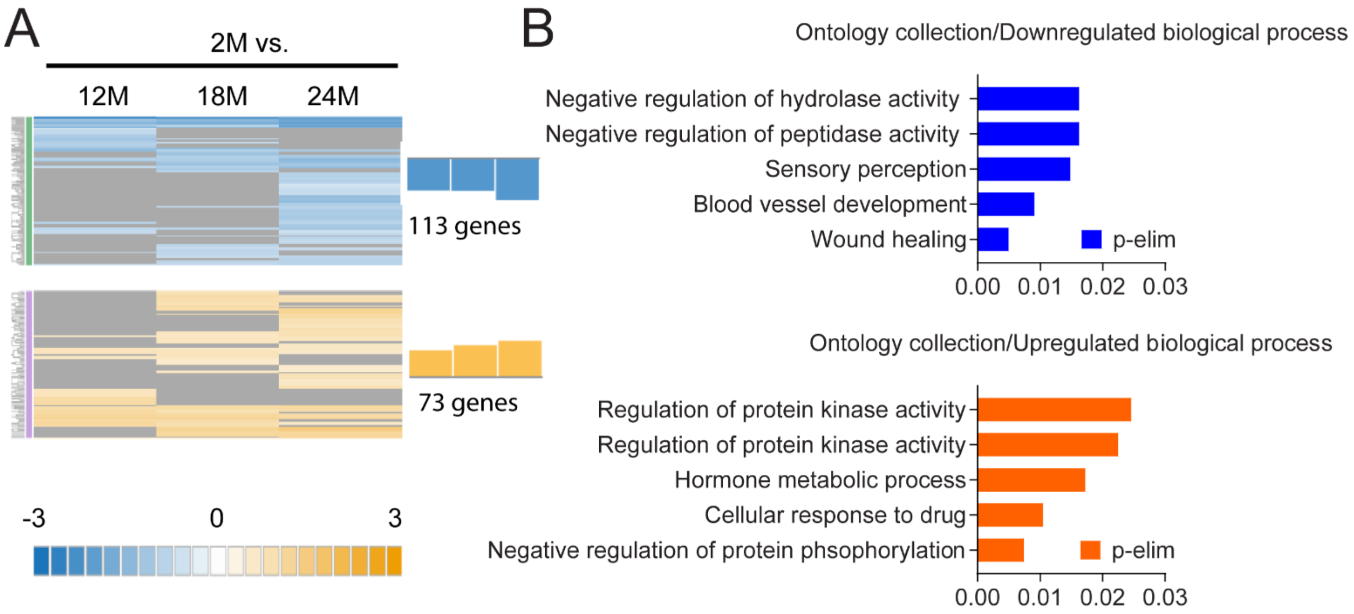

Supplemental Figure 2. **ROSALIND Metanalysis of DEGs comparing the different ages. A.** Overall graph representation of metanalysis showing downregulated (blue) or upregulated (orange) genes. Genes that did not pass the FDR are shown in grey. **B**. Gene Ontology Collection showing down and upregulated pathways.

Supplemental Figure 3

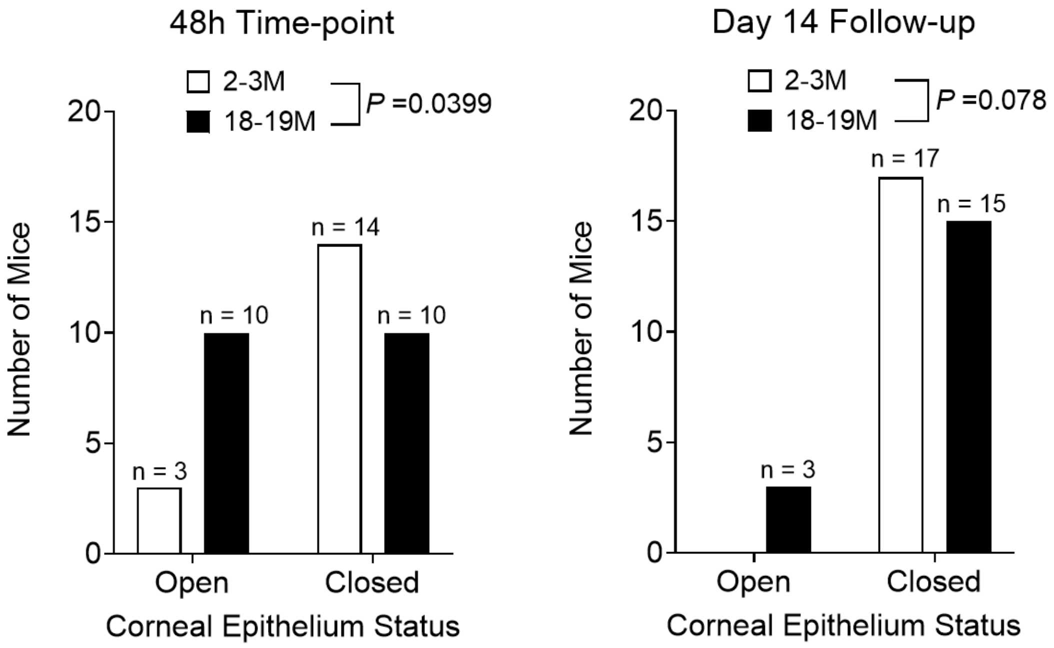

Supplemental Fig. 3. Follow up on corneal epithelium 48 hours and 14 days post corneal debridement. A. Cumulative data of corneas 48 hours post-debridement. Older mice significantly showed more unhealed corneas than the young. Chi Square test was used to analysis the data. M = months, n = number. **B**. Cumulative data of corneas 14 days post-debridement. Chi Square test was used to analysis the data. M = months, n = number.
